# Molecular Epidemiology of Japanese Encephalitis Virus in Mosquitoes in Taiwan during 2005–2012

**DOI:** 10.1371/journal.pntd.0003122

**Published:** 2014-10-02

**Authors:** Chien-Ling Su, Cheng-Fen Yang, Hwa-Jen Teng, Liang-Chen Lu, Cheo Lin, Kun-Hsien Tsai, Yu-Yu Chen, Li-Yu Chen, Shu-Fen Chang, Pei-Yun Shu

**Affiliations:** 1 Center for Research, Diagnostics and Vaccine Development, Centers for Disease Control, Ministry of Health and Welfare, Taiwan, Republic of China; 2 Institute of Environmental Health, College of Public Health, National Taiwan University, Taipei, Taiwan; 3 Department of Public Health, College of Public Health, National Taiwan University, Taipei, Taiwan; 4 Infectious Diseases Research and Education Center, Ministry of Health and Welfare and National Taiwan University, Taipei, Taiwan, Republic of China; University of Texas Medical Branch, United States of America

## Abstract

Japanese encephalitis (JE) is a mosquito-borne zoonotic disease caused by the Japanese encephalitis virus (JEV). Pigs and water birds are the main amplifying and maintenance hosts of the virus. In this study, we conducted a JEV survey in mosquitoes captured in pig farms and water bird wetland habitats in Taiwan during 2005 to 2012. A total of 102,633 mosquitoes were collected. *Culex tritaeniorhynchus* was the most common mosquito species found in the pig farms and wetlands. Among the 26 mosquito species collected, 11 tested positive for JEV by RT-PCR, including *Cx. tritaeniorhynchus*, *Cx. annulus*, *Anopheles sinensis*, *Armigeres subalbatus*, and *Cx. fuscocephala*. Among those testing positive, *Cx. tritaeniorhynchus* was the predominant vector species for the transmission of JEV genotypes I and III in Taiwan. The JEV infection rate was significantly higher in the mosquitoes from the pig farms than those from the wetlands. A phylogenetic analysis of the JEV envelope gene sequences isolated from the captured mosquitoes demonstrated that the predominant JEV genotype has shifted from genotype III to genotype I (GI), providing evidence for transmission cycle maintenance and multiple introductions of the GI strains in Taiwan during 2008 to 2012. This study demonstrates the intense JEV transmission activity in Taiwan, highlights the importance of JE vaccination for controlling the epidemic, and provides valuable information for the assessment of the vaccine's efficacy.

## Author Summary

Japanese encephalitis (JE) is a vector-borne zoonotic disease transmitted by the bite of a Japanese encephalitis virus (JEV) infected mosquito. Japanese encephalitis is an endemic disease in Taiwan. Before 2008, all known JEV isolates collected in Taiwan belonged to Genotype III of JEV. Genotype I JEV strains were first found in northern Taiwan in 2008. In this study, we conducted a survey of JEV in mosquitoes during 2005–2012. A total of 102,633 mosquitoes were collected from pig farms and wetlands. Among the 26 mosquito species collected, 11 tested JEV positive by RT-PCR, including *Cx. tritaeniorhynchus*, *Cx. annulus* and *An. sinensis*. *Cx. tritaeniorhynchus* was the predominant vector species for transmission of JEV in Taiwan. The JEV infection rate of the mosquitoes captured on the pig farms was significantly higher than the rate of those captured in the wetlands, indicating that pigs played an important role in amplifying JEV. A phylogenetic analysis of the envelope gene sequences of JEV isolated from the mosquitoes demonstrated that the predominant JEV genotype shifted from genotype III to genotype I (GI), providing evidence for multiple introductions and transmission cycle maintenance of GI strains in Taiwan during 2008–2012.

## Introduction

Japanese encephalitis is a vector-borne zoonotic disease transmitted by the bite of a JEV-infected mosquito. Although JE is a vaccine preventable disease, JEV infections are still the leading cause of viral encephalitis in Asia [1], [2]. It is estimated that 67,900 JE cases occur annually in JE endemic countries, with an incidence rate of 1.8 cases per 100,000 individuals [3]. Symptoms of JE include fever, chills, headache, myalgia, weakness, mental disturbances and neurologic symptoms. The mortality rate can reach as high as 30%, and approximately 30–50% of survivors suffer severe neurological damage [4], [5].

The JEV belongs to the genus *Flavivirus* of the family Flaviviridae and is a single-stranded positive-sense RNA virus. The viral genome is approximately 11 kb in length and encodes three structural proteins [capsid (C), premembrane (prM), and envelope (E)], followed by 7 non-structural proteins (NS1, NS2A, NS2B, NS3, NS4A, NS4B, NS5) [6], [7]. According to phylogenetic analysis of the E protein gene sequences, the JEV strains can be classified into 5 distinct genotypes (genotypes I–V) [8], [9]. Recent studies suggested that the epidemic/dominant genotype of JEV has gradually shifted from genotype III (GIII) to genotype I (GI) in Southeast and East Asian countries in the last two decades [10]–[14].

Japanese encephalitis is an endemic disease in Taiwan and has been designated as a notifiable infectious disease since 1955. The highest incidence rate of confirmed JE cases (2.05 per 100,000) was recorded in 1967. After the mass JE vaccination program was implemented in 1968, the incidence rate of confirmed JE cases declined significantly [15]–[17]. From 1998 to 2011, the annual number of confirmed JE cases ranged from 13 to 37. In 2012, 32 JE cases were confirmed, which is equivalent to an incidence rate of 0.13 per 100,000 individuals. From 1998 to 2012, the epidemic peak months were June and July. Confirmed cases occurred sporadically throughout Taiwan. Most individuals diagnosed with JE lived near rice paddy fields or pig farms [18].

We previously reported the molecular epidemiology of JEV in Taiwan [19]. The study demonstrated that all known JEV isolates collected before 2008 belonged to GIII. Genotype I JEV strains that were first found in northern Taiwan in 2008. In the present study, we monitored the dynamics of the genotype transition and genetic variation of JEV and identified the mosquito species potentially involved in the transmission of JEV in Taiwan during 2005–2012.

## Materials and Methods

### Mosquito collections

**Figure 1:**
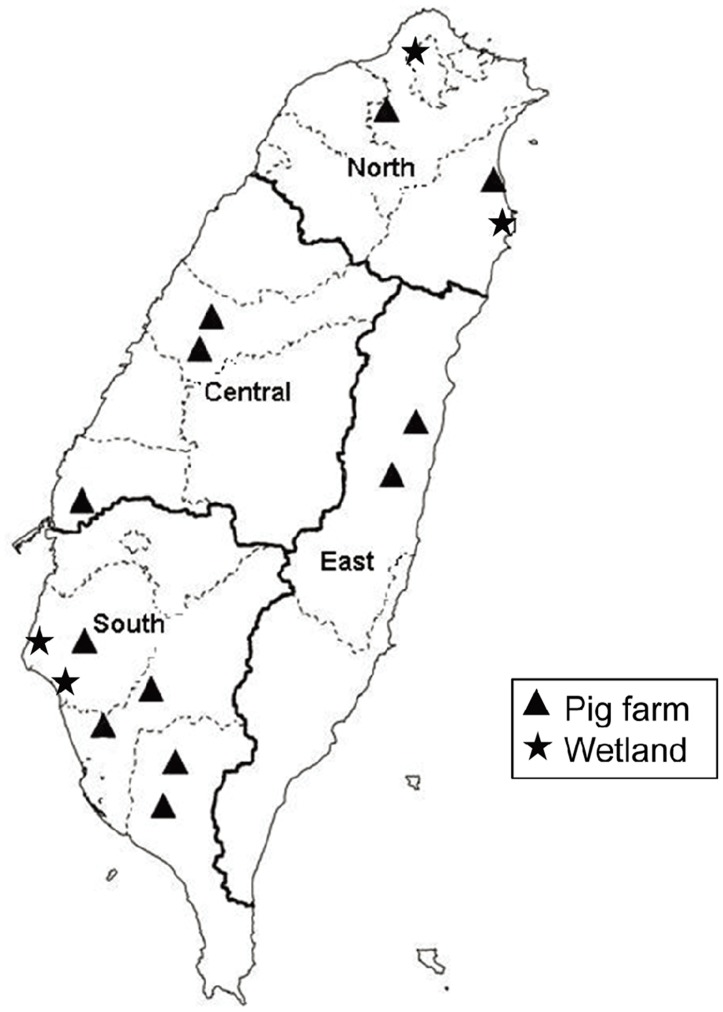
Map showing mosquito collection sites in northern, central, southern and eastern Taiwan. Mosquitoes collected from pig farms and wetlands are indicated with a triangle (▴) and star (★), respectively.

Mosquitoes were collected on pig farms near rice paddy fields in the northern (Yingge District in New Taipei City and Wujie Township in Yilan County), central (Beitun and Wufeng Districts in Taichung City and Shuilin Township in Yunlin County), southern (Xiaying District in Tainan City, Neimen and Alian Districts in Kaohsiung City, Yanpu and Zhutian Townships in Pingtung County), and eastern (Shoufeng and Guangfu Townships in Hualein County) regions of Taiwan and from wetland habitats for water-birds in the northern (Beitou District in Taipei City, and Su'ao Township in Yilan County) and southern (Qigu and Anping Districts in Tainan City) regions from 2005 to 2012 (Figure 1). The mosquitoes were collected using dry ice traps or sweep nets and were transported either alive or on dry ice to the laboratory. Mosquito collections by sweep nets were conducted only on the same day between 18:30 and 20:30 on the pig farms, while dry ice traps were set up overnight from 17:30 to 7:30 the next morning in the pig farms and wetlands. The predominant mosquito species collected by dry ice trap and sweep net were the same at each collection site. Since dry ice trap method had a long duration of time for mosquito collection, more mosquitoes in numbers and species were captured with this method. The mosquitoes were pooled by species, sex, location, and collection date in groups of 1–50 mosquitoes. Only female mosquitoes were analyzed in this study. The mosquito pools were homogenized in a TissueLyzer (Qiagen GmbH, Hilden, Germany) with two cycles at 4°C for 90 sec at a frequency of 30 Hz after adding a 3 mm steel ball to each tube. The pools were then clarified by centrifugation. The supernatants were then sterilized by filtration and removed for RNA extraction and virus isolation.

### RNA extraction and real time RT-PCR

Viral RNA was extracted from the mosquito suspensions using the QIAamp viral RNA mini kit (Qiagen). Three sets of primers, including flavivirus-specific (FL-F1: 5′-GCCATATGG TACATGTGGCTGGGAGC-3′; FL-R3: 5′-GTKATTCTTGTGTCCCAWCCGGCTGTGTCATC-3′; FL-R4: 5′-GTGATGCGRGTGTCCCAGCCRGCKGTGTCATC-3′), JEV-specific (JE3F1: 5′-CCCTCAGAACCGTCTCGGAA-3′ and JE3R1: 5′-CTATTCCCAGGTGTCAATATGCTGT-3′) and JEV GIII-specific (10F: 5′-CTGGGAATGGGCAATCGTG-3′ and 5′-TGTCAATGCTTCCCTTCCC-3′) primers, were used for the RT-PCR assay [19], [20]. Real-time RT-PCR was used to screen for JEV in the mosquito pools as previously described [21]. DNA sequences of positive RT-PCR products were determined. JEV positive samples were then subjected to virus isolation.

### Virus isolation and genome sequencing

Cell culture techniques using a mosquito C6/36 cell line or plaque assay using the BHK-21 cell line were used for virus isolation as described previously [22]. Viral RNA was extracted from the JEV-infected culture medium using the QIAamp viral RNA mini kit (Qiagen). The primers used for the amplification and sequencing of the complete open reading frame of JEV are listed in Table 1. The RT-PCR reaction was carried out using the Superscript III One-Step RT-PCR system with Platinum Taq High Fidelity (Invitrogen). The RT-PCR reaction was performed under the following parameters: 55°C for 30 min; 94°C for 2 min; 40 cycles of 94°C for 15 sec, 50°C for 30 sec, and 68°C for 1 min; and a prolonged elongation at 68°C for 5 min. RT-PCR products were purified using the Qiagen QIA quick Gel Extraction kit (QIAGEN). Nucleotide sequences were determined using the ABI Prism automated DNA sequencing kit and the ABI Prism 3700 DNA sequencer (Applied Biosystems) according to the manufacturer's protocols. Overlapping nucleotide sequences were combined and edited using the Lasergene software package (DNASTAR Inc., Madison, WI). Nucleotide sequences of JEV strains were aligned, edited, and analyzed using ClustalW software. The phylogenetic analysis was performed using MEGA 5 (http://www.megasoftware.net/) [23]. The phylogenetic tree was generated using the maximum likelihood method based on the general time-reversible model. The reliability of the analysis was calculated using 1,000 bootstrap replications. The nucleotide sequences of 50 JEV strains isolated from the mosquitoes and a strain isolated from a human were submitted to Genbank with the following accession numbers: KF667277–KF667327.

**Table 1:** Primers used in RT-PCR and DNA sequencing for Japanese encephalitis virus (JEV).

Primer name	Sequence 5′-3′	Primer used
JE-5UTRF	AGA AGT TTA TCT GTG TGA ACT TCT TGG	PCR, sequencing
JE-616R	CCT CAC ACA TGT AGC CGA CGT CT	Sequencing
JE-747R	TTC GCT TGG AAT GCC TGG TCC G	Sequencing
JE-747F	CGG ACC AGG CAT TCC AAG CGA A	Sequencing
JE-1448R	GGA AGC ATT GAC ACA TGT GCA AAA TT	PCR, sequencing
JE-1309F	AGA ACA ATC CAG CCA GAA AAC ATC	PCR, sequencing
JE-1360F	CGC TGA ATA ATT CCC ATG GTT TTC	Sequencing
JE-1839F	AGG CTG AAA ATG GAC AAA CTG GC	Sequencing
JE-1878R	GGT TGT GCC TTT CAG AGC CAG TTT	Sequencing
JE-2515R	ATC TCT TTT CTT GTG ATG TCA ATG GC	Sequencing
JE-2602R	AGG GAT CTG GGC GTT TCT GG	Sequencing
JE-2636R	GCC TTC CTT GTG CGC TTT GT	PCR, sequencing
JE-2340F	GGG AAT GTC TTG GAT CAC ACA AGG	PCR, sequencing
JE-2926F	TGG AAC AGC ATG CAA ATC GAA GA	Sequencing
JE-3032R	CCT ATG ATC GCT CCA TCA CAC TC	Sequencing
JE-3630R	GGT ACG GAA TGG AAA TCA GAC CTG	PCR, sequencing
JE-3467F	CAA TCT GGC CGT CCA CCT CTT GC	PCR, sequencing
JE-4169F	CCA CTA TAG CTG CCG GAC TAA TGG	Sequencing
JE-4324R	CTG CCA GCA TGA AGG GTA TTG AC	Sequencing
JE-4946R	TGG CAC ACA ACT AGA GGA GCA GC	PCR, sequencing
JE-4756F	GGT TTT GTC TGG ATG TTT ACT GC	PCR, sequencing
JE-4946F	GCA GTA AAC ATC CAG ACA AAA CC	Sequencing
JE-5424R	TGA CAT CAG TCT ATG GGT CAG AGT	Sequencing
JE-5424F	ACT CTG ACC CAT AGA CTG ATG TCA	Sequencing
JE-6096R	CCA TCC CCC ATA ACC AGT GCA AG	PCR, sequencing
JE-5946F	TAA CAT GAT CTT TGC CTC TGT CC	PCR, sequencing
JE-6096F	GGA CAG AGG CAA AGA TCA TGT TA	Sequencing
JE-6678F	GAT GCA GCG AAA GGG TAT AGG GAA	Sequencing
JE-6770R	GTT CCA GGA ACC TCT GCC GCC CA	Sequencing
JE-7388R	TGG ATG GCA AGC AGA AGC ACT CAG	PCR, sequencing
JE-7293F	ACA TCA GTG GCG ACC ATT CCG TC	PCR, sequencing
JE-7469F	CAT AGG GGT AAG CGT GGC AGC GTT	Sequencing
JE-7829F	GAG GAC ATC CGG TTT CGC GAG	Sequencing
JE-8030R	CTC TGC ATG AGC ATC GGT TCT TC	Sequencing
JE-8579R	GCG AAT GGA TCG CAC AGT GTG GAG	PCR, sequencing
JE-8403F	GGA TGC TCA GGG TCT TTG TGC CA	PCR, sequencing
JE-8579F	TGG CAC AAA GAC CCT GAG CAT CC	Sequencing
JE-9185R	GAA CAG AAT CAA TGG AGC ACA GC	Sequencing
JE-8926F	TCT CGG CTC AGC CAA TGG TCT TC	PCR, sequencing
JE-9185F	GAA GAC CAT TGG CTG AGC CGA GA	PCR, sequencing
JE-9719R	TCA AGA GAA GAC CAA AGG GGG AGT	Sequencing
JE-10266R	ATG GCG ATC AGC GGA GAC GAC T	PCR, sequencing
JE-9489F	CTG TGT CGT CAA GCC GCT GG	PCR, sequencing
JE-9664F	GTG GCG AAT CTG TCG TCC AGC GG	Sequencing
JE-9703F	GTG GTC ATC CAC TCT CCT TTC GAG	Sequencing
JE-10086R	CCA GAT GTC CTC ACG CTT TCC CAC	Sequencing
JE-10230R	GGT TGC TCT GGA TCG CGT TCC GAT	Sequencing
JE-10266F	ATC GGA ACG CGA TCC AGA GCA ACC	Sequencing
JE-10488F	ACT GGG TAG ACG GTG CTG CCT G	Sequencing
JE-10980R	AGA TCC TGT GTT CTT CCT CAC CAC	PCR, sequencing

### Infection rates in mosquitoes and statistical analysis

The maximum likelihood estimates (MLEs) of the mosquito JEV infection rate in mosquitoes were calculated using the PooledInfRate software by Biggerstaff (www.cdc.gov/ncidod/dvbid/westnile/software.htm) [24]. The Chi-squared test was used for comparison of the mosquito JEV infection rates of different species and sampling sites.

## Results

### Mosquito species and infection rates of JEV

Mosquitoes were captured from 16 localities in Taiwan from 2005 to 2012 (Figure 1). A total of 102,633 mosquitoes belonging to the family Culicidae were collected and analyzed. Table 2 shows a summary of the mosquito species, the MLE of the JEV infection rate per 1,000 mosquitoes and the mosquito collection sites (all localities, pig farms, and wetlands). Twenty-six mosquito species from 8 genera of the Culicidae family were identified. The most frequently identified species was *Cx. tritaeniorhynchus* Giles (86.90%, n = 89,189), followed by *Cx. sitiens* Wiedemann (6.13%, n = 6,295) and *Anopheles sinensis* Wiedemann (2.57%, n = 2,638). Of the 2,848 mosquito pools subjected to real-time RT-PCR for the detection of JEV, 499 were positive. The most frequently identified JEV positive mosquito species was *Cx. tritaeniorhynchus* (468 positive pools), followed by *Cx. annulus* (9 positive pools) and *An. sinensis* (6 positive pools); the MLEs of the JEV infection rates per 1,000 individuals in these three species were 5.85, 8.99, and 2.3, respectively. There were significant differences between JEV infection rates in *Cx. tritaeniorhynchus* and *An. sinensis* at p<0.05 (P = 0.0357), and in *Cx. annulus* and *An. sinensis* at p<0.01 (P = 0.0044), but was no significant difference in *Cx. tritaeniorhynchus* and *Cx. annulus* at p<0.05 (P = 0.0979). *Culex tritaeniorhynchus* and *An. sinensis* were the most frequently identified mosquito species on the pig farms, whereas *Cx. tritaeniorhynchus* and *Cx. sitiens* were the most frequent in the wetlands. The MLE for the mosquito JEV infection rate was significantly higher on the pig farms (7.5 per 1000) than in the wetlands (1.73 per 1000) (p<0.01).

**Table 2:** Summary of the mosquito species, the maximum likelihood estimates (MLEs) of the mosquito Japanese encephalitis virus (JEV) infection rate, and the mosquito collection sites (all localities, pig farms, and wetlands).

	All localities		Pig farms		Wetlands	
Species	MLE (95% CI)^3^	No. positive pools/No. pools/No. individuals^4^	MLE (95% CI)	No. positive pools/No. pools/No. individuals	MLE (95% CI)	No. positive pools/No. pools/No. individuals
*Aedes aegypti* 1	0 (0.00–499.14)	0/2/3	0.00 (0.00–499.14)	0/2/3	-	-
*Ae. albopictus*	5.38(0.33–25.36)	1/25/177	19.44 (1.30–88.53)	1/12/46	0.00 (0.00–22.75)	0/13/131
*Ae. penghuensis* 2	0 (0.00–10.46)	0/10/283	-	-	0.00 (0.00–10.46)	0/10/283
*Ae. vexans*	12.85 (3.49–34.75)	3/32/246	29.65 (1.91–139.58)	1/7/32	9.75 (1.79–32.33)	2/25/214
*Anopheles ludlowae* 1	0 (0.00–793.45)	0/1/1	0.00 (0.00–793.45)	0/1/1	-	-
*An. minimus* 1	52.78 (3.38–230.59)	1/7/18	52.78 (3.38–230.59)	1/7/18	-	-
*An. sinensis*	2.30 (0.95–4.73)	6/119/2638	2.05 (0.77–4.50)	5/104/2464	5.33 (0.34–25.44)	1/15/174
*An. tessellatus*	3.66 (0.68–11.74)	2/31/536	4.81 (0.33–23.37)	1/7/180	2.75 (0.16–13.24)	1/24/356
*Armigeres subalbatus*	13.49 (3.69–35.78)	3/30/225	17.34 (4.83–45.72)	3/22/175	0.00 (0.00–56.26)	0/8/50
*Coquillettidia crassipes* 2	0 (0.00–35.54)	0/3/47	-	-	0.00 (0.00–35.54)	0/3/47
*Culex annulus*	8.99 (4.67–15.91)	9/79/991	26.29 (13.89–46.52)	8/46/301	1.41 (0.08–6.76)	1/33/690
*Cx. bitaeniorhynchus* 2	0 (0.00–37.88)	0/7/60	-	-	0.00 (0.00–37.88)	0/7/60
*Cx. brevipalpis* 2	0 (0.00–793.45)	0/1/1	-	-	0.00 (0.00–793.45)	0/1/1
*Cx. fuscanus*	0 (0.00–450.75)	0/3/4	0.00 (0.00–499.14)	0/2/3	0.00 (0.00–793.45)	0/1/1
*Cx. fuscocephala* 1	7.77 (2.17–20.82)	3/19/394	7.77 (2.17–20.82)	3/19/394	-	-
*Cx. mimeticus* 2	0 (0.00–793.45)	0/1/1	-	-	0.00 (0.00–793.45)	0/1/1
*Cx. murrelli* 2	0 (0.00–53.12)	0/3/39	-	-	0.00 (0.00–53.12)	0/3/39
*Cx. nigropunctatus* 2	0 (0.00–160.75)	0/1/9	-	-	0.00 (0.00–160.75)	0/1/9
*Cx. quinquefasciatus*	1.51 (0.27–4.95)	2/74/1333	1.64 (0.30–5.38)	2/67/1226	0.00 (0.00–26.51)	0/7/107
*Cx. rubithoracis* 2	0 (0.00–42.44)	0/8/65	-	-	0.00 (0.00–42.44)	0/8/65
*Cx. sitiens* 2	0 (0.00–0.60)	0/128/6295	-	-	0.00 (0.00–0.60)	0/128/6295
*Cx. tritaeniorhynchus*	5.85 (5.34–6.40)	468/2242/89189	7.68 (6.97–8.45)	413/1653/61765	2.10 (1.60–2.72)	55/589/27424
*Mansonia uniformis*	13.82 (0.79–68.46)	1/19/75	18.14 (1.06–90.71)	1/12/57	0.00 (0.00–146.56)	0/7/18
*Ochlerotatus albolateralis* 2	0 (0.00–793.45)	0/1/1	-	-	0.00 (0.00–793.45)	0/1/1
*Oc. togoi* 2	0 (0.00–793.45)	0/1/1	-	-	0.00 (0.00–793.45)	0/1/1
*Uranotaenia macfarlanei* 1	0 (0.00–793.45)	0/1/1	0.00 (0.00–793.45)	0/1/1	-	-
Total	5.35 (4.90–5.83)	499/2848/102633	7.50 (6.82–8.22)	439/1962/66666	1.73 (1.33–2.21)	60/886/35967

1 Mosquito species captured at pig farms only.

2 Mosquito species captured in wetlands only.

3 The maximal likelihood estimation per 1000 mosquitoes and the 95% confidence interval (lower limit-upper limit).

4 The number of JEV positive pools/the number of pools tested/the number of mosquitoes tested by RT-PCR.

### Mosquito infection rates of JEV by month

**Figure 2:**
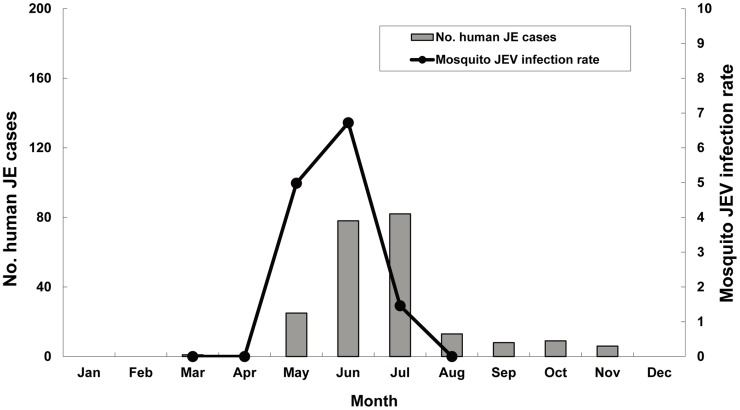
Japanese encephalitis epidemic season in humans and MLE of the JEV infection rates in mosquitoes by month during 2005–2012.

Table 3 shows the MLEs of the JEV infection rate per 1,000 mosquitoes by month and regions. JEV infected mosquitoes first appeared in early May, peaked in June, and then declined in July. The MLEs for the infection rates for May, June, and July were 4.98, 6.72, and 1.46, respectively. May was the peak month in the southern and eastern regions, whereas June was the peak month in the northern and central regions. Figure 2 shows the mosquito JEV infection rates and the numbers of confirmed human JE cases per month during 2005–2012. Spring and summer were the epidemic seasons of JE in Taiwan, and June and July were the peak months for human JE cases.

**Table 3:** Summary of the maximum likelihood estimates (MLEs) of the mosquito Japanese encephalitis virus (JEV) infection rate by month, and the mosquito collection sites in the northern, central, southern, eastern, and all sites of Taiwan between 2005 and 2012.

Month/Region	All sites	Northern	Central	Southern	Eastern
Mar	0 (0.00–65.51); 0/10/44^1^	ND^2^	ND	0 (0.00–89.55); 0/5/27	0 (0.00–139.30); 0/5/17
Apr	0 (0.00–1.05); 0/110/3586	0 (0.00–94.07); 0/4/24	0 (0.00–15.37); 0/12/207	0 (0.00–1.19); 0/80/3134	0 (0.00–14.67); 0/14/221
May	4.98 (4.13–5.97); 113/696/24948	3.66 (2.43–5.31); 25/214/7272	4.45 (2.35–7.75); 11/77/2682	2.46 (1.62–3.60); 24/265/10220	14.66 (11.10–19.15); 53/140/4774
Jun	6.72 (6.07–7.42); 370/1599/61889	7.09 (6.27–8.01); 249/1017/39692	7.80 (6.33–9.52); 92/337/13772	0.95 (0.39–1.98); 6/175/6382	12.08 (8.14–17.44); 23/70/2043
Jul	1.46 (0.87–2.32); 16/385/11180	1.16 (0.43–2.57); 5/146/4422	2.47 (1.27–4.40); 10/137/4157	0 (0.00–1.69); 0/61/2186	2.35 (0.14–11.24); 1/41/415
Aug	0 (0.00–3.71); 0/48/986	0 (0.00–19.15); 0/13/166	0 (0.00–12.93); 0/14/259	0 (0.00–6.22); 0/21/561	ND
Total	5.35 (4.90–5.83); 499/2848/102633	6.01 (5.34–6.74); 279/1394/51576	6.02 (4.99–7.22); 113/577/21077	1.37 (0.94–1.93); 30/607/22510	12.32 (9.82–15.32); 77/270/7470

1 The maximal likelihood estimation of the JEV infection rate per 1000 mosquitoes and the 95% confidence interval (lower limit-upper limit); and the number of JEV positive pools/the number of pools tested/the number of mosquitoes tested by RT-PCR.

2 Not done.

### Mosquito infection rates of JEV in different geographical regions

Table 4 shows the MLEs of the mosquito JEV infection rates in different geographical locations. In general, the JEV infection rate was highest in the eastern region (12.32 per 1000) (p<0.01), followed by central (6.02 per 1000) and northern (6.01 per 1000) regions, and lowest in the southern region (1.37 per 1000) (p<0.01).

**Table 4:** Summary of the maximum likelihood estimates (MLEs) of the mosquito Japanese encephalitis virus (JEV) infection rate per year, and the mosquito collection sites (all sites, northern, central, southern, and eastern regions).

	All sites		Northern		Central		Southern		Eastern	
Year/Region	IR (95%CI); No. positive pools/No. pools/No. individuals^1^	No. GI/No. GIII^2^	IR (95%CI); No. positive pools/No. pools/No. individuals	No. GI/No. GIII	IR (95%CI); No. positive pools/No. pools/No. individuals	No. GI/No. GIII	IR (95%CI); No. positive pools/No. pools/No. individuals	No. GI/No. GIII	IR (95%CI); No. positive pools/No. pools/No. individuals	No. GI/No. GIII
2005	12.12 (10.57–13.85); 206/742/20020	0/99	16.31 (13.70–19.31); 128/340/9810	0/39	6.06 (4.32–8.30); 36/218/6478	0/26	6.66 (3.87–10.83); 15/97/2562	0/15	27.68 (19.10–39.27); 27/87/1170	0/19
2006	5.35 (3.91–7.17); 42/236/8659	0/42	10.32 (7.17–14.52); 31/92/3613	0/31	3.84 (1.97–6.86); 10/73/2785	0/10	0 (0.00–3.70); 0/24/960	0/0	0.77 (0.04–3.75); 1/47/1301	0/1
2007	0.59 (0.11–1.93); 2/87/3410	0/2	5.04 (0.30–25.91); 1/7/202	0/1	0 (0.00–2.93); 0/31/1240	0/0	0 (0.00–2.81); 0/32/1288	0/0	1.47 (0.09–7.20); 1/17/680	0/1
2008	11.04 (8.08–14.81); 42/159/4454	2/40	19.45 (13.24–27.96); 28/64/1894	2/26	7.10 (2.93–14.86); 6/35/921	0/6	3.38 (1.10–8.14); 4/43/1229	0/4	11.02 (3.63–27.09); 4/17/410	0/4
2009	2.05 (1.60–2.60); 65/801/33039	14/33	1.77 (1.27–2.40); 38/548/22326	6/15	3.56 (1.76–6.54); 9/61/2699	8/0	0.16 (0.01–0.80); 1/143/6064	0/1	10.48 (6.39–16.50); 17/49/1950	0/17
2010	3.27 (2.50–4.22); 56/482/18385	46/10	4.31 (2.60–6.78); 17/118/4355	17/0	7.13 (4.39–11.09); 18/75/2924	11/7	0.60 (0.25–1.24); 6/260/10097	3/3	23.17 (13.34–39.37); 15/29/1009	15/0
2011	6.01 (4.21–8.37); 33/146/6314	33/0	3.08 (1.72–5.15); 13/107/4522	13/0	15.87 (9.99–24.63); 20/39/1792	20/0	ND		ND	
2012	7.54 (5.71–9.81); 53/195/8352	51/2	5.36 (3.49–7.94); 23/118/4854	21/2	7.35 (4.22–12.12); 14/45/2238	14/0	17.00 (5.76–45.24); 4/8/310	4/0	18.57 (10.15–33.02); 12/24/950	12/0
Total	5.35 (4.90–5.83); 499/2848/102633	146/228	6.01 (5.34–6.74); 279/1394/51576	59/114	6.02 (4.99–7.22); 113/577/21077	53/49	1.37 (0.94–1.93); 30/607/22510	7/23	12.48 (9.97–15.50); 77/270/7470	27/42

1 The maximal likelihood estimation of the JEV infection rate per 1000 mosquitoes and the 95% confidence interval (lower limit-upper limit); and the number of JEV positive pools/the number of pools tested/the number of mosquitoes tested by RT-PCR.

2 Numbers of Genotype I and genotype III JEV positive pools determined by RT-PCR and DNA sequencing.

### Genotype shifting of JEV in Taiwan

**Figure 3:**
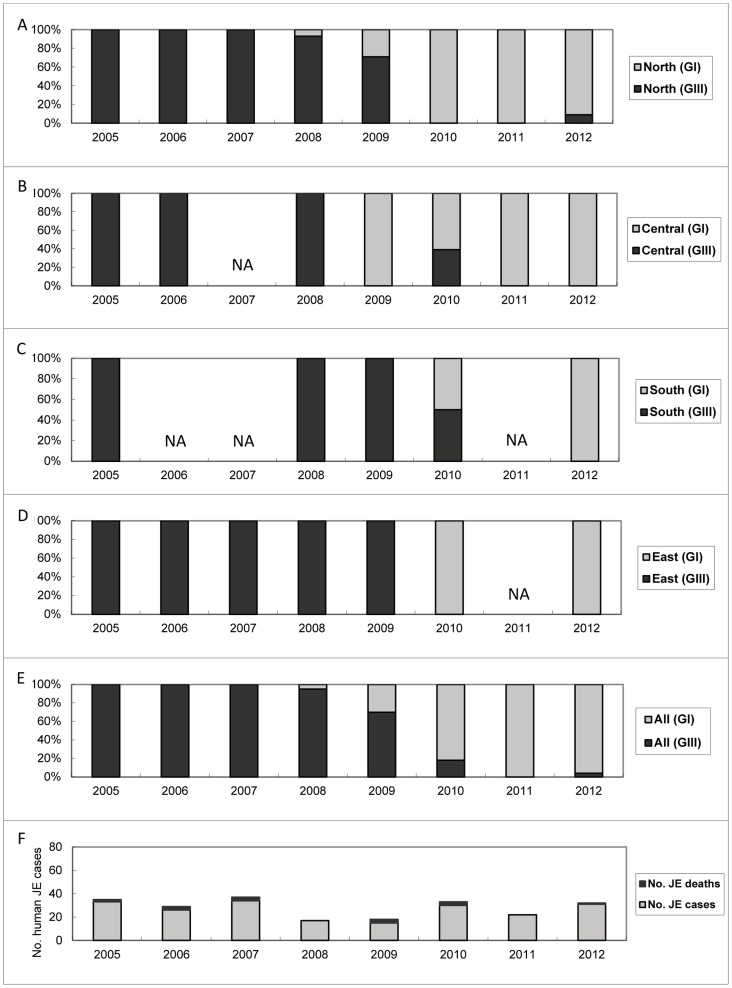
Proportions of genotype distributions of Japanese encephalitis virus strains and annual numbers of Japanese encephalitis cases in Taiwan between 2005 and 2012. The proportions of genotype distributions in northern (A), central (B), southern (C), eastern (D), all (E) of Taiwan, and the annual numbers of confirmed and death cases of individuals with Japanese encephalitis (F). NA = not available.

Table 4 also shows the numbers of GI and GIII JEV positive pools determined by DNA sequencing of RT-PCR products per year. Of the 499 mosquito pools that were JEV positive by real-time RT-PCR, 374 pools were genotyped by sequencing the real time-RT-PCR products. The real-time RT-PCR was performed using two sets of primers, one primer set targeting a region of the nonstructural protein 5 (NS5) genes to detect all of the flaviviruses, and the other primer set targeting a region of the 3′ untranslated region (3′UTR) to detect JEV. Both partial NS5 gene sequence (154 bp) and 3′UTR sequence (220 bp) contain sufficient information to differentiate genotypes of JEV. All of the JEV positive pools were genotyped each year, except in 2005 and 2009, only representative RT-PCR positive samples were selected (based on the place and date of collections) for sequencing and genotyping. Figure 3A–3E shows the proportional distribution of the GI and GIII JEV strains identified in the northern, central, southern, eastern, and all sites of Taiwan between 2005 and 2012. The annual numbers of RT-PCR positive pools for genotype analysis were 99, 42, 2, 42, 47, 56, 33 and 53, respectively, during 2005 to 2012 (Table 4). Before 2008, all the JEV found in Taiwan belonged to GIII. GI was first identified in northern Taiwan in 2008. Since then, the proportion of GI isolates in Taiwan has increased rapidly. From 2009 to 2010, GI became the predominant JEV genotype circulating in Taiwan. Since 2011, almost all of the JEV isolates obtained in Taiwan have belonged to GI, with the exception of 2 GIII strains found in Kuantu Nature Park in Taipei City in 2012. Because GIII was the only JEV genotype identified in 2005–2007 and GI was the only genotype found in 2011 (Table 5), to estimate the mosquito JEV infection rates according to genotype, we compared the difference between the JEV infection rates in 2005–2007 and 2011. The GIII JEV infection rate per 1,000 mosquitoes in 2005–2007 was 8.86, and the GI JEV infection rate was 6.01 in 2011, there was no significant difference between JEV infection rates in these two groups at p<0.01 (P = 0.0294, chi-squared test). Although JEV infection rates for both GIII and GI were not the highest in *Cx. tritaeniorhynchus*, this mosquito species was the most dominant species harboring the JEV. Among 374 JEV RT-PCR positive pools that were genotyped, 89.0% (203/228) of GIII and 96.6% (141/146) of GI were *Cx. tritaeniorhynchus* mosquitoes. The GIII JEV infection rate per 1,000 mosquitoes of *Cx. tritaeniorhynchus* was 8.90 in 2005–2007, and the GI rate was 6.26 in 2011, there was no significant difference between JEV infection rates in these two groups (P = 0.0514, chi-squared test).

**Table 5:** Summary of the mosquito species, the maximum likelihood estimates (MLEs) of the mosquito Japanese encephalitis virus (JEV) infection rates by year.

Species	2005	2006	2007	2005–2007^1^	2008	2009	2010	2011	2012
*Aedes aegypti*							0 (0.00–499.14); 0/2/3^2^		
*Ae. albopictus*	30.48(2.28–138.68); 1/6/27			30.48 (2.28–138.68); 1/6/27		0.00 (0.00–97.86); 0/5/27	0 (0.00–25.12); 0/12/110	0 (0.00–545.52); 0/1/2	0 (0.00–133.58); 0/1/11
*Ae. penghuensis*							0 (0.00–10.46); 0/10/283		
*Ae. vexans*	31.04 (2.05–147.76); 1/5/30	0 (0.00–793.45); 0/1/1		30.34 (1.98–143.52); 1/6/31	0 (0.00–793.45); 0/1/1	14.92 (2.86–49.98); 2/16/141	0 (0.00–112.36); 0/3/19	0 (0.00–103.70);; 0/3/20	0 (0.00–60.73); 0/3/34
*Anopheles ludlowae*							0 (0.00–793.45); 0/1/1		
*An. minimus*	66.36 (4.87–294.18); 1/4/13	0 (0.00–499.14); 0/2/3		58.09 (3.84–252.43); 1/6/16			0 (0.00–545.52); 0/1/2		
*An. sinensis*	4.22 (1.61–9.21); 5/50/1207	0 (0.00–8.02); 0/13/409		3.14 (1.19–6.86); 5/63/1616	0 (0.00–13.09); 0/12/248	2.37 (0.14–11.37); 1/20/407	0 (0.00–10.98); 0/18/292	0 (0.00–33.34); 0/5/74	0 (0.00–793.45); 0/1/1
*An. tessellatus*	NA^3^; 1/1/1			NA; 1/1/1		3.47 (0.21–16.68); 1/20/281	0 (0.00–12.47); 0/8/233	0 (0.00–99.81); 0/1/15	0 (0.00–231.16); 0/1/6
*Armigeres subalbatus*	26.12 (7.62–68.18); 3/15/116			26.12 (7.62–68.18); 3/15/116	0 (0.00–178.93); 0/1/8	0 (0.00–122.60); 0/5/21	0 (0.00–42.11); 0/8/68		0 (0.00–123.16); 0/1/12
*Coquillettidia crassipes*						0 (0.00–35.21); 0/1/44			0 (0.00–499.14); 0/2/3
*Culex annulus*	21.49 (9.04–48.19); 4/18/169	22.91 (1.49–112.23); 1/5/41		22.91 (10.22–47.10); 5/23/210	111.48 (27.79–316.17); 2/5/15	1.52 (0.09–7.28); 1/27/639	10.68 (0.70–49.65); 1/14/85	0 (0.00–66.85); 0/5/28	0 (0.00–165.40); 0/5/14
*Cx. bitaeniorhynchus*						0 (0.00–200.11); 0/5/12	0 (0.00–145.91); 0/1/10		0 (0.00–40.66); 0/1/38
*Cx. brevipalpis*							0 (0.00–793.45); 0/1/1		
*Cx. fuscanus*						0 (0.00–657.62): 0/2/2	0 (0.00–545.52); 0/1/2		
*Cx. fuscocephala*	13.01 (3.96–47.66); 2/5/110			13.01 (3.96–47.66); 2/5/110	14.86 (1.08–138.51); 1/2/64	0 (0.00–20.65); 0/5/129	0 (0.00–29.48); 0/7/91		
*Cx. mimeticus*									0 (0.00–793.45); 0/1/1
*Cx. murrelli*						0 (0.00–793.45); 0/1/1	0 (0.00–61.14); 0/1/25		0 (0.00–114.25); 0/1/13
*Cx. nigropunctatus*									0 (0.00–160.75); 0/1/9
*Cx. quinquefasciatus*	4.58 (0.84–15.05); 2/24/445	0 (0.00–40.88); 0/5/51		4.10 (0.75–13.47); 2/29/496	0 (0.00–16.35); 0/12/195		0 (0.00–6.17); 0/30/566	0 (0.00–31.32); 0/3/76	
*Cx. rubithoracis*	0 (0.00–58.86); 0/1/26			0.00 (0.00–58.86); 0/1/26		0 (0.00–300.42); 0/2/6	0 (0.00–173.58); 0/2/11		0 (0.00–103.51); 0/3/22
*Cx. sitiens*						0 (0.00–2.09); 0/37/1745	0 (0.00–0.83); 0/91/4550		
*Cx. tritaeniorhynchus*	12.41 (10.73–14.30); 185/607/17843	5.59 (4.07–7.52); 41/208/8144	0.59 (0.11–1.93); 2/87/3410	8.90 (7.81–10.11); 228/902/29397	11.84 (8.56–16.06); 39/126/3923	2.21 (1.64–2.72); 60/651/29578	5.13 (3.91–6.63); 55/266/12011	6.26 (4.39–8.72); 33/126/6096	7.73 (5.85–10.05); 53/171/8184
*Mansonia uniformis*	32.92 (1.89–173.90)	0 (0.00–161.60); 0/2/10		24.72 (1.42–126.77); 1/8/43		0 (0.00–367.00); 0/3/5	0 (0.00–99.47); 0/3/20	0 (0.00–499.14); 0/2/3	0 (0.00–450.75); 0/3/4
*Ochlerotatus albolateralis*							0 (0.00–793.45); 0/1/1		
*Oc. togoi*						0 (0.00–793.45); 0/1/1			
*Uranotaenia macfarlanei*							0 (0.00–793.45); 0/1/1		
Total	12.12 (10.57–13.85); 206/742/20020	5.35 (3.91–7.17); 42/236/8659	0.59 (0.11–1.93); 2/87/3410	8.86 (7.82–10.00); 250/1065/32089	11.04 (8.08–14.81); 42/159/4454	2.05 (1.60–2.60); 65/801/33039	3.27 (2.50–4.22); 56/482/18385	6.01 (4.21–8.37); 33/146/6314	7.54 (5.71–9.81); 53/195/8352

1 Combined data from the 2005 to 2007.

2 The maximal likelihood estimation of the JEV infection rate per 1000 mosquitoes and the 95% confidence interval (lower limit-upper limit); and the number of JEV positive pools/the number of pools tested/the number of mosquitoes tested by RT-PCR.

3 Not available.

### Phylogenetic analysis of JEV isolated from mosquitoes

**Figure 4:**
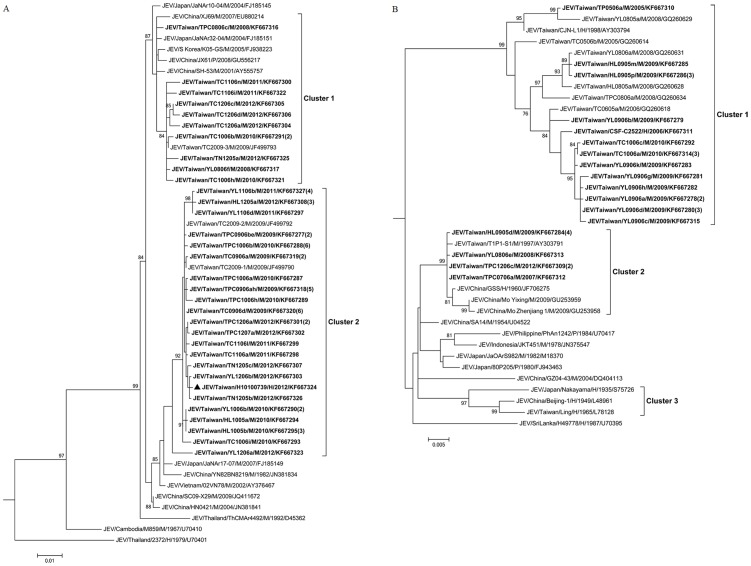
Phylogenetic analysis of Japanese encephalitis virus. The phylogenetic tree shows the genetic relationship of genotype I (A) and genotype III (B) of Japanese encephalitis virus isolates. The tree was constructed based on the nucleic acid sequences of complete envelope (E) genes of the JEV strains. The sequences obtained in this study are indicated in **boldface**. A sequence obtained from a human case is indicated with a triangle (▴). Viruses were identified by using the nomenclature of virus/country/strain/source/year of isolation/GenBank accession number. Numbers in parentheses indicate the number of isolates that showed 100% nucleotide homology. Virus isolates with the same sequences were collected at the same time from the same locations in this study. CH = Changhua County; HL = Hualien County; KH = Kaohsiung City; TC = Taichung City; TN = Tainan City; TP = New Taipei City; TPC = Taipei City; TY = Taoyuan County; YL = Yilan County; M = mosquito pool; P = pig serum; H = human sample. Analysis was performed using MEGA 5 software and the maximum likelihood method based on the general time-reversible model. The reliability of the analysis was calculated using 1,000 bootstrap replications. Bootstrap support values>75 are shown. The scale bar indicates nucleotide substitutions per site.

A total of 148 JEV isolates (147 from *Cx. tritaeniorhynchus* and one from *Cx. annulus*) were obtained by virus isolation, and the complete E gene sequences of these isolates were determined. In this study, 50 E gene sequences covered the entire sequence diversity of JEV in Taiwan were selected for phylogenetic analysis. The JEV strains isolated from mosquitoes in Taiwan during 2005–2012 fell into two genotypes (GI and GIII). Figure 4A shows the phylogenetic tree of the E gene sequences of GI, which can be grouped into 2 clusters. Cluster 1 contains the JEV strains isolated from mosquitoes collected throughout the country during 2008–2012, including the first two GI isolates (TPC0806c and YL0806f) identified in Taiwan. The GI virus strains in Cluster 2 were first identified in northern and central Taiwan in 2009; in the following year, these strains were found throughout Taiwan. Cluster 2 also contained a JEV strain isolated from a patient (H10100739) who lived in Kaohsiung City. These strains are most closely related to the viruses from China and Japan. These results indicate that multiple introductions and transmission cycle maintenance of the GI strains occurred during 2008–2012.

Figure 4B shows the phylogenetic tree of GIII JEV. The strains isolated in Taiwan between 2005 and 2012 were divided into 2 clusters (Clusters 1 and 2). The majority of the GIII strains isolated in Taiwan during 2005 to 2011 were classified as Cluster 1. However, in our study, no strains belonging to this lineage were found in 2012. Cluster 2 of GIII included a minor group of JEV strains in Taiwan. Most of the isolates were found in the northern and eastern parts of Taiwan. In 2012, only 2 isolates (TPC1206c-1, TPC1206c-2) belonging to GIII JEV were found in Taiwan. The Cluster 2 strains of GIII are closely related to viruses from China, Japan, Indonesia and the Philippines.

## Discussion

In this study, we reported the results of a survey of JEV-infected mosquitoes from pig farms and wetlands in Taiwan during 2005 to 2012. Pig farms near rice paddy fields and wetland habitats for water birds are common in Taiwan, and these places provide suitable environments for the JEV infection cycle [25]. Confirmed JE cases have been identified throughout Taiwan with most of the infected individuals residing near pig farms or rice paddy fields [18]. *Cx. tritaeniorhynchus* was the predominant mosquito species captured from both the pig farms (92.6%) and the wetlands (76.2%) and was the main species infected with JEV genotypes I and III. Un-baited sweep net sampling method is considered a passive method that can be used to collect a wide variety of mosquito species. Dry ice (CO_2_)-baited trap can attract host-seeking female mosquitoes, and CO_2_ appears to be universally attractive to a variety of mosquito species [26]. Therefore, these two mosquito sampling methods do not seem to have a collection bias towards *Cx. tritaeniorhynchus* or any of the other mosquito species. These results provide evidence that *Cx. tritaeniorhynchus* remains a principal vector for the transmission of JEV in Taiwan. Although other *Culex* mosquitoes, such as *Cx. annulus* and *Cx. fuscocephala* Theobald, were also reported as important JEV vectors [27]–[29], a relatively low number of these mosquitoes were captured, indicating that they play a minor role in the transmission of JEV in Taiwan.

The JEV infection rate of mosquitoes captured on the pig farms (7.50 per 1000) was significantly higher than the rate of those captured in the wetlands (1.73 per 1000) (p<0.01), indicating that pigs played an important role in amplifying JEV. In addition, except for *A. sinensis*, the infection rates of all of mosquito species collected on the pig farms were higher than the rates of those in the wetlands (Table 2). In our study, JEV positive mosquitoes were captured in only one of the four wetlands, located in Beitou District, Taipei City. This wetland is near human habitats, where both water birds and pigs may serve as reservoirs or amplifying hosts for the JEV.

Interestingly, although 11 species of mosquitoes were RT-PCR positive for JEV, the virus was isolated solely from *Cx. tritaeniorhynchus* and *Cx. annulus*. Because both blood-fed and unfed mosquitoes were analyzed, and because the RT-PCR results did not allow differentiation between mosquitoes that were actually infected with JEV from those with residual virus in the blood meals, the MLEs of the JEV infection rates in mosquitoes may have been overestimated in this study.

According to the Taiwan CDC's surveillance data, the JE epidemic season has occurred annually between May and October, peaking between June and July in recent decades. These results are in accordance with our mosquito surveillance report, where JEV positive mosquito pools appeared in early May, peaked in June, and then disappeared in July. Because most pigs raised for food on pig farms are not immunized with JEV vaccine, only pigs used for breeding are vaccinated in Taiwan, the rapid decline of the JEV infection rates in the mosquitoes captured on the pig farms might be due to an increase in the JEV antibody positive rates of the pigs. In addition, a relatively low JEV positive mosquito rate during this period was observed due to the high mosquito density between July and September [30] in Taiwan.

Before 2008, all the JEV strains identified in Taiwan belonged to GIII. We first found GI JEV in northern Taiwan in 2008, since then, virus strains of this genotype have rapidly spread throughout the country [19], [31]. During 2011–2012, nearly all the JEV strains found in Taiwan belonged to GI. However, although the JEV genotype shifted dramatically from GIII to GI, no obvious change was found in the annual numbers of confirmed JE cases during this period (Figure 3F), suggesting that the JEV vaccination was still effective against newly introduced GI strains in Taiwan.

In our study, the JEV E gene sequence phylogenetic analysis provided evidence for multiple introductions and maintenance of the transmission cycles of the GI strains in Taiwan (Figure 4A). Ecological factors, such as climate and landscape, may influence the geographic distribution of the JEV genotypes [29], [32]. Taiwan is an island located in the Western Pacific off the southeast coast of China, and the Tropic of Cancer passes through the central part of the island. The climate is warm, rain-water is abundant, and many of the wetlands provide suitable habitats for mosquito vectors and water birds. In addition, because pork and rice are the main agricultural products in Taiwan, pig farms and rice paddy fields are very common in the suburban areas of Taiwan. Now that the new GI strains have been introduced to the ideal transmission environment of Taiwan, the new viruses may be able to establish their transmission cycles in new territories. Gao et al. [14] recently reported that the southernmost region of Asia (Thailand, Vietnam, and Yunnan Province, China) may have been the source of GI JEV transmission to the Asian continent including Taiwan. The Clusters 1 and 2 of the GI JEV strains in Taiwan belonged to the lineages of the eastern coastal Asian endemic cycle and the central Asian endemic cycle, respectively, suggesting that the GI JEV strains were most likely introduced from China and Japan to Taiwan in recent years. In our study, we found that GI JEV first appeared in northern Taiwan in 2008. In the following year, GI strains were found in northern and central Taiwan. Subsequently, these viruses spread across Taiwan (Figure 3A–D). The direction of GI JEV transmission in Taiwan seems to be in accordance with the transmission mode proposed by Gao et al. [14]. However, the reasons why the GI strains replaced the GIII strains within such a short period of time, and the ecological and biological factors involved in this event, are still unclear. Further studies are needed to address these questions.

This study demonstrated the intense JEV transmission activity in Taiwan and highlights the importance of JE vaccination to control this epidemic. Continuous monitoring of the JEV strain variations and their gene sequence evolution can provide valuable information for the assessment of the vaccine's efficacy.
